# A mid-level health manager intervention to promote uptake of isoniazid preventive therapy among people with HIV in Uganda: a cluster randomised trial

**DOI:** 10.1016/S2352-3018(22)00166-7

**Published:** 2022-07-28

**Authors:** Elijah Kakande, Canice Christian, Laura B Balzer, Asiphas Owaraganise, Joshua R Nugent, William DiIeso, Derek Rast, Jane Kabami, Jason Johnson Peretz, Carol S Camlin, Starley B Shade, Elvin H Geng, Dalsone Kwarisiima, Moses R Kamya, Diane V Havlir, Gabriel Chamie

**Affiliations:** Infectious Diseases Research Collaboration, Kampala, Uganda; Department of Medicine, University of California, San Francisco, San Francisco, CA, USA; Department of Biostatistics and Epidemiology, University of Massachusetts, Amherst, MA, USA; Infectious Diseases Research Collaboration, Kampala, Uganda; Department of Biostatistics and Epidemiology, University of Massachusetts, Amherst, MA, USA; Sustainable East Africa Research Collaboration (SEARCH)-IPT Trial, Mbarara, Uganda; Sustainable East Africa Research Collaboration (SEARCH)-IPT Trial, Mbarara, Uganda; Infectious Diseases Research Collaboration, Kampala, Uganda; Department of Medicine, University of California, San Francisco, San Francisco, CA, USA; Department of Medicine, University of California, San Francisco, San Francisco, CA, USA; Department of Medicine, University of California, San Francisco, San Francisco, CA, USA; Department of Medicine, Washington University in St Louis, St Louis, MO, USA; Infectious Diseases Research Collaboration, Kampala, Uganda; Infectious Diseases Research Collaboration, Kampala, Uganda; Department of Medicine, Makerere University, Kampala, Uganda; Department of Medicine, University of California, San Francisco, San Francisco, CA, USA; Department of Medicine, University of California, San Francisco, San Francisco, CA, USA

## Abstract

**Background:**

Despite longstanding guidelines endorsing isoniazid preventive therapy (IPT) for people with HIV, uptake is low across sub-Saharan Africa. Mid-level health managers oversee IPT programmes nationally; interventions aimed at this group have not been tested. We aimed to establish whether providing structured leadership and management training and facilitating subregional collaboration and routine data feedback to mid-level managers could increase IPT initiation among people with HIV compared with standard practice.

**Methods:**

We conducted a cluster randomised trial in Uganda among district-level health managers. We randomly assigned clusters of between four and seven managers in a 1:1 ratio to intervention or control groups. Our intervention convened managers into mini-collaboratives facilitated by Ugandan experts in tuberculosis and HIV, and provided business leadership and management training, SMS platform access, and data feedback. The control was standard practice. Participants were not masked to trial group, but study statisticians were masked until trial completion. The primary outcome was IPT initiation rates among adults with HIV in facilities overseen by participants over a period of 2 years (2019–21). We conducted prespecified analyses that excluded the third quarter of 2019 (Q3–2019) to understand intervention effects independent of a national 100-day IPT push tied to a financial contingency during Q3–2019. This trial is registered with ClinicalTrials.gov (NCT03315962), and is ongoing.

**Findings:**

Between Nov 15, 2017, and March 14, 2018, managers from 82 of 82 eligible districts (61% of Uganda’s 135 districts) were enrolled and randomised: 43 districts to intervention, 39 to control. Intervention delivery took place between Dec 6, 2017, and Feb 2, 2022. Over 2 years, IPT initiation rates were 0·74 versus 0·65 starts per person-year in intervention versus control groups (incidence rate ratio [IRR] 1·14, 95% CI 0·88–1·46; p=0·16). Excluding Q3–2019, IPT initiation was higher in the intervention group versus the control group: 0·32 versus 0·25 starts per person-year (IRR 1·27, 95% CI 1·00–1·61; p=0·026).

**Interpretation:**

Following an intervention targeting managers in more than 60% of Uganda’s districts, IPT initiation rates were not significantly higher in intervention than control groups. After accounting for large increases in IPT from a 100-day push in both groups, the intervention led to significantly increased IPT rates, sustained after the push and during the COVID-19 pandemic. Our findings suggest that interventions centred on mid-level health managers can improve IPT implementation on a large, subnational scale, and merit further exploration to address key public health challenges for which strong evidence exists but implementation remains suboptimal.

**Funding:**

National Institute of Allergy and Infectious Diseases.

## Introduction

Isoniazid preventive therapy (IPT) reduces the risk of active tuberculosis by approximately 40–60% for people with HIV,^1^ in addition to tuberculosis risk reduction from antiretroviral therapy.^2^ Although IPT has been recommended by WHO for all people with HIV in high tuberculosis burden settings since 2008, increasing IPT uptake across sub-Saharan Africa has remained a challenge.^3^ Multiple barriers have been reported, including concerns about ruling out active tuberculosis before starting IPT, isoniazid resistance in tuberculosis disease post-IPT, and insufficient health-care worker knowledge.^4^ However, over the past decade, WHO and country guidelines have provided simple clinical algorithms for ruling out active tuberculosis among people with HIV to facilitate IPT uptake,^5^ and several studies have shown the lack of association of IPT with increased isoniazid resistance.^6,7^ Nonetheless, IPT use has remained below the international goal of reaching all people with HIV who are unlikely to have active tuberculosis.^8^ Although 3·5 million people with HIV received tuberculosis preventive therapy globally in 2019, India, South Africa, and Tanzania accounted for 56% of the total, and only 1·5 million people with HIV received tuberculosis preventive therapy throughout the rest of sub-Saharan Africa^3^—a region home to two-thirds of the estimated 37·7 million people with HIV globally.^9^

Health-care middle managers oversee implementation of guidelines at the subnational level in many sub-Saharan African countries. In Uganda, a country with a high burden of tuberculosis and HIV in which less than 2% of people with HIV had received IPT by 2018,^10^ the Ministry of Health (MoH) provides strategic direction by developing guidelines, and 135 district-level managers lead guideline implementation and manage service delivery, including health-care workers. Because each manager oversees budgetary, educational, and operational aspects of service delivery for a catchment area of hundreds of thousands of residents per district, interventions targeting these managers offer novel opportunities to achieve rapid impacts at a national scale. However, middle managers typically lack formal leadership or management training. Therefore, we conducted a cluster randomised controlled trial to establish whether an intervention centred on district-level managers that provided structured leadership and management training and facilitated subregional collaboration and routine data feedback could increase IPT initiation among people with HIV compared with standard practice in three regions of Uganda.

## Methods

### Study design and participants

We conducted a cluster randomised controlled trial in Uganda that enrolled district-level managers: district health officers, the highest-ranking MoH leaders in each district; and tuberculosis supervisors, who oversee tuberculosis-specific activities and report to health officers. Each district in Uganda has one district health officer and one tuberculosis supervisor. The trial compared an intervention for managers to increase IPT initiation for adults with HIV against standard practice (control). We used a cluster randomised design because the intervention was delivered to groups of managers. The Makerere University School of Medicine Research and Ethics Committee, the Uganda National Council for Science and Technology, and the University of California San Francisco Committee on Human Research approved the study protocol.

We recruited all district-level managers from the southwestern, east-central, and eastern regions of Uganda in 2017 (southwestern) and 2018 (east-central and eastern). All participants provided written informed consent. We created 14 clusters (between four and seven districts per cluster), based on geographical adjacency, number of urban versus rural districts, number of people with HIV in care, and region. Clusters were pair-matched on characteristics expected to be predictive of IPT initiation: region, number of adults in HIV care, presence of large urban centres, and presence of a community that had participated from 2013 to 2017 in the SEARCH HIV test-and-treat trial (NCT01864603).

### Randomisation and masking

Within each pair, the clusters were randomly assigned in a 1:1 ratio to intervention or control groups at region-specific participatory meetings where representatives of each cluster selected envelopes revealing trial groups when opened. There were two sealed, opaque envelopes, identical in appearance for each matched pair: one with a paper inside with “intervention” on it, and one with a paper inside with “control” on it. Representatives from each group came to the front of the room, and at the same time, selected an envelope, opening them simultaneously to reveal (to the room) the group assignment. Clusters were not masked to randomisation group, but study statisticians (LBB, JRN) were masked until trial completion. Periodically, the Government institutes district divisions. When this occurred, we maintained randomisation status of the original district for newly formed districts and offered enrolment to new managers. We initiated trial data collection on Dec 6, 2017, and closed data collection on June 30, 2021.

### Procedures

Our study intervention centred on managers and used the PRECEDE framework for health promotion strategies to address “predisposing factors” (knowledge, attitudes, or beliefs that affect behaviour); “enabling factors” that make a behaviour easier; and “reinforcing factors” that include anticipated consequences following a behaviour.^11^ We selected intervention components on the basis of their theoretical capacities to change behaviour in support of IPT initiation, and feedback from a pre-trial focus group among district health officers in southwestern Uganda (unpublished data).

Our intervention first convened each cluster of managers into a “mini-collaborative”, informed by the Institute for Healthcare Improvement Breakthrough series.^12^ The collaboratives met biannually, with an additional meeting 2–3 months after the first, to increase uptake of up-to-date information and positive attitudes towards IPT. Small-world network theory suggests that random links between mini-collaborative members will speed diffusion of predisposing information and attitude change.^13^ Theories of social influence and persuasion suggest that, because of their authority and influence, attitude change among managers will change practice among the frontline health-care workers they oversee.^14^ Each mini-collaborative meeting was facilitated by a Ugandan expert in tuberculosis and HIV from national referral medical centres or research organisations.

Second, we offered intensive 1-day, interactive courses in leadership and management skills during mini-collaborative meetings to enable IPT use. Each course was designed and led by two international business professionals and our study team, with courses emphasising specific tools to improve leadership and management skills, adapted to a Ugandan context. The first course focused on Kotter’s eight-step model for leading change in organisations,^15^ the second on using “Objectives and Key Results”^16^ and the third on “Start/Stop/Continue” team feedback (appendix pp 2–5).^17^

We offered access to a two-way text messaging (SMS) system to facilitate communication and data inquiries between managers and frontline workers. In each district, two tuberculosis point persons (frontline health workers at clinics who are the key contacts for tuberculosis data questions with the district managers) had the option to provide weekly tuberculosis reports to managers via toll-free SMS messaging. The report included metrics on tuberculosis screening, IPT prescribing, tuberculosis diagnoses, and isoniazid stocks.

Lastly, at biannual meetings we provided up-to-date data dashboards that included each mini-collaborative’s quarterly progress in number of adults with HIV initiating IPT, proportion of eligible adults with HIV initiated on IPT, isoniazid stock availability, and active tuberculosis case counts at the two largest clinics per district. Managers also received their district-specific metrics. We obtained data for the dashboards from the MoH. We used dashboards to provide comparisons among managers within and across mini-collaboratives, thereby using a reputational approach to reinforce IPT.

The control group of the trial involved standard practice. Following randomisation, we ensured that control-group managers received Uganda MoH IPT guidelines.^18^ MoH support for IPT implementation in all districts included training at the time of IPT guideline release in 2014 and access to isoniazid, upon request and when available, via Uganda’s National Medical Stores. Districts also had access to an MoH and UNICEF-supported one-way SMS system that allowed clinics to send weekly reports on IPT to MoH, for review during performance review meetings, held at the discretion of each district’s manager. Throughout the duration of the trial, MoH guidelines recommended IPT for all adults with HIV without symptoms suggestive of active tuberculosis, including pregnant women. The guidelines also specified IPT contraindications, including specific medications (eg, warfarin) or comorbidities (eg, liver disease). The managers in the control group were organised into clusters for purposes of cluster randomisation only.

At baseline, 1 year, and 2 years post-randomisation, we conducted a brief survey among participating managers in both trial groups regarding knowledge and perceived attitudes towards IPT and tuberculosis prevention among people with HIV, communication practices, and perceptions of influence on health-care workers. For each district and year, we calculated the average response to five-point Likert scale questions (appendix p 8).

Among intervention participants, we conducted focus group discussions (FGDs) to elicit perceptions of intervention content and impact. Southwest FGDs were held at 1 year and 2 years post-randomisation. FGDs in the east and east-central regions were held only 1 year post-randomisation, as the COVID-19 pandemic prevented 2-year follow-up. In total, we conducted four FGDs with between seven and 11 managers per FGD. Key informant interviews (KIIs) were conducted with between four and eight randomly selected control-group managers per region (23 KIIs; similar timepoints as FGDs) to understand facilitators of and barriers to IPT in control districts.

We collected data to estimate incremental annual cost, from the implementor perspective, of the intervention overall and by district. We interviewed study staff to identify resources consumed and amount of time dedicated to planning and conducting mini-collaborative meetings. Costs associated with business professional travel, lodging, and donated time were calculated and included. Before selected trainings, we administered surveys for managers to estimate time required to implement intervention activities during the previous 6 months.

### Outcomes

The primary outcome was the incidence of IPT initiation, defined as the rate in person-years at which adults (15 years or older) with HIV received an IPT prescription in health facilities overseen by managers participating in the trial. This endpoint was measured over eight quarters (starting in the first quarter of 2019 [Q1–2019] for the southwest region, and Q2–2019 for the east and east-central regions) at the two largest clinics in each district. Before unmasking, we prespecified the measurement period to begin in 2019 to account for national isoniazid stock-outs during 2018, described in the following section. For each clinic, quarterly data on the number of IPT starts and number of adults in HIV care were extracted from the MoH Health Management Information System (HMIS) database. We calculated the incidence rate over 2 years of follow-up as the total number of starts divided by the total person-time-at-risk of IPT initiation at the two largest clinics in each district (appendix p 8). In prespecified secondary analyses, we evaluated the 2-year cumulative incidence of IPT initiation, calculated as total number of IPT starts divided by average active HIV care population size at the two largest clinics in each district. When measuring IPT initiation, we did not account for IPT ineligibility, including prevalent tuberculosis, due to incomplete reporting of ineligibility data within HMIS and based on the assumption that IPT ineligibility would be balanced by trial group due to randomisation and shared national guidelines for IPT.

Secondary outcomes included IPT completion, incidence rate of HIV-associated tuberculosis, and changes in IPT knowledge and management skills among managers. Another secondary outcome (specified in the protocol) was frontline provider assessments of the leadership and management of their supervisors, which we will report in a separate, future manuscript. IPT completion was defined as documented refill of a 6-month IPT course within 9 months of initiation. As measures of IPT completion were not available in HMIS, we conducted a chart review of isoniazid refills among a subset of 800 randomly selected patients (400 per trial group) who were aged 15 years or older, living with HIV, and started IPT at one of 16 facilities (eight per trial group) in the southwest region. To account for the national 100-day IPT push (Q3–2019), described in the following section, chart review sampling was stratified on IPT initiation time, with half starting before Q3–2019 and half during Q3–2019.

We defined HIV-associated tuberculosis incidence as the rate at which people in active HIV care were diagnosed with tuberculosis disease. District-level data were extracted from HMIS for all study districts and incidence rates calculated analogously to the primary outcome.

Changes in IPT knowledge and management skills were assessed through a survey among participating managers, and through FGDs (intervention group) and KIIs (control group), as described in the previous section.

### Statistical analysis

Based on standard calculations,^19^ we estimated that 14 clusters would provide 80% power to detect a 12% or greater absolute increase in IPT initiation from 22 per 100 person-years under the control, assuming a coefficient of variation of 0·25 and around 21 500 person-years of follow-up in each cluster.

In the primary analysis, we compared district-level IPT incidence rates in an intention-to-treat analysis using targeted minimum loss-based estimation (TMLE), an approach that is appropriate for cluster randomised trials and adaptively selects the optimal adjustment variables to maximise precision, while preserving type-I error control.^20,21^ Specifically, we used leave-one-out cross-validation to select from the following set of prespecified candidates: baseline IPT uptake, baseline active care size, or nothing (unadjusted). With the Student’s *t*-distribution, we calculated two-sided 95% CIs and tested the null hypothesis that the intervention did not improve IPT uptake compared with control, with a one-sided test at the 5% significance level (appendix p 8).

To estimate the intervention effect on HIV-associated tuberculosis disease risk, we compared district-level tuberculosis incidence rates by group using an analogous approach as for the primary outcome. For evaluation of changes in response to quantitative surveys, we compared district-level responses by group with TMLE.

Among the 800 sampled participants for IPT completion, we evaluated the group-specific and relative risk of IPT completion with an individual-level TMLE, adjusting for sex, age, and timing of IPT initiation, and tested the null hypothesis of no improvement from the intervention (one-sided test at the 5% significance level).

All analyses accounted for the cluster randomised design (appendix p 8) and were conducted in R version 4.0.3. An overview of how district data and participant subgroups were included in primary and secondary analyses is provided (appendix p 9).

For qualitative analyses, we used the rigorous and accelerated data reduction technique on transcripts from KIIs to understand control-group manager attitudes, and from FGDs to uncover perceptions of intervention impact.^22^ Results were coded along four categories: impact of (1) study overall, (2) data dashboards, (3) leadership and management courses, and (4) intervention on non-IPT activities.

Three major secular events occurred during the trial, resulting in modifications to measures and analyses before unblinding, in accordance with CONSERVE 2021 recommendations (appendix p 6).^23^ First, in 2017–18, Uganda experienced isoniazid stock-outs nationwide, that improved by early 2019. To test our intervention in a context where isoniazid supply was not the limiting factor, we prespecified our endpoint measurement period over 2 years starting in 2019 (Q1–2019 southwest region, and Q2–2019 east and east-central regions). Second, the MoH, with support from the US President’s Emergency Plan for AIDS Relief (PEPFAR), instituted a 100-day IPT push (“100-day IPT scale-up plan”) in Q3–2019 described in their strategic plan to include training of health providers, isoniazid stock, and IPT initiation targets to select facilities, to increase IPT nationwide, with a target of 300 000 people with HIV initiated on tuberculosis preventive therapy for Q3–2019.^24^ PEPFAR’s 2019 Country Operation Plan for Uganda indicated that “TPT [tuberculosis preventive therapy] for all PLHIV [people living with HIV] must be scaled-up as an integral and routine part of the HIV clinical care package”, with a target of 400 000 people with HIV in 2019, as one of “the minimum requirements for continued PEPFAR support”.^25^ To better understand our intervention’s impact on IPT initiation independent of the 100-day push, we conducted prespecified secondary analyses that excluded Q3–2019. Finally, in Q2–2020, the Ugandan President ordered a nationwide COVID-19 lockdown. We conducted prespecified sensitivity analyses that evaluated outcomes pre-lockdown and post-lockdown.

This trial is registered with ClinicalTrials.gov (NCT03315962).

### Role of the funding source

The funder of the study had no role in study design, data collection, data analysis, data interpretation, or writing of the report.

## Results

Of 79 eligible districts in Uganda invited to participate in the trial at baseline, managers from all districts agreed to participate. Randomisation was performed at participatory events on Nov 15, 2017 (southwest), Feb 28, 2018 (east), and March 14, 2018 (east-central). Seven clusters (between five and seven districts per cluster; 40 districts total) were randomly assigned to intervention and seven clusters (between four and seven districts per cluster; 39 districts total) to control. Following district divisions in July, 2019, there were 43 districts in the intervention group and 40 in the control group. One newly formed district in the control group was excluded from analysis due to lack of data on primary and secondary outcomes.

**Figure 1:**
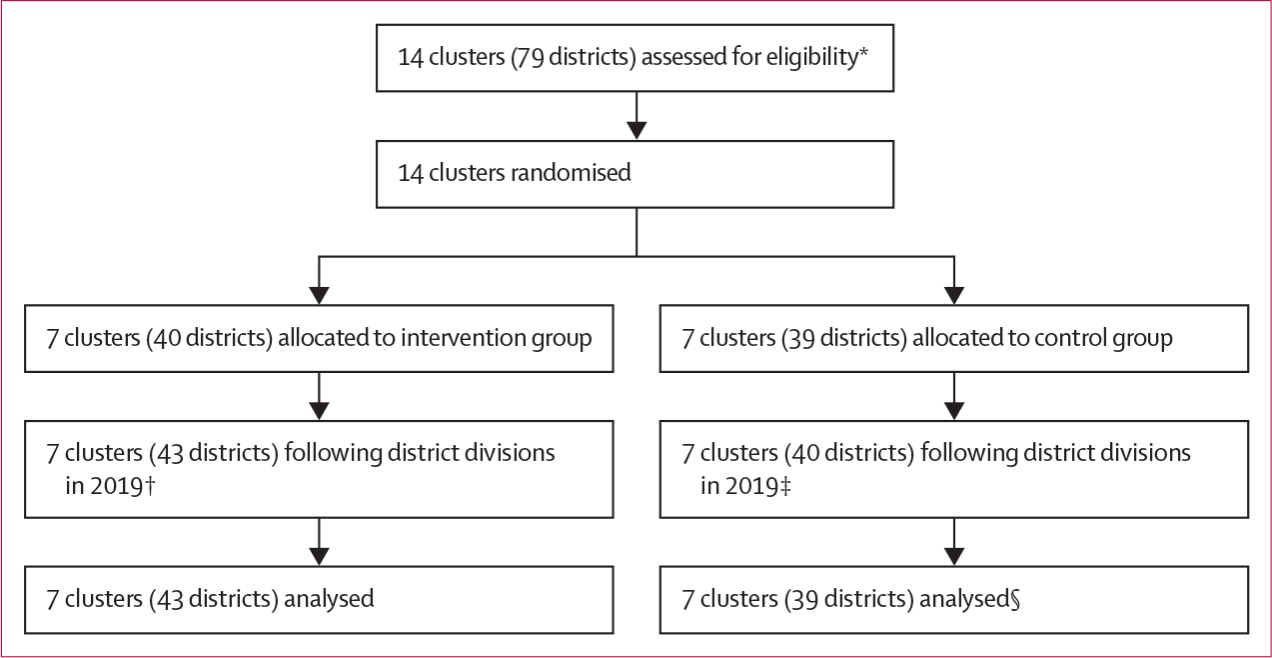
Trial profile *Two managers (one district health officer and one tuberculosis supervisor) per district. In one district, one manager (district health officer) declined to participate, but this manager’s district was represented by the tuberculosis supervisor. †Three districts were divided, creating three new districts. ‡One district was divided, creating one new district. §One district was excluded due to lack of data for primary and secondary outcomes.

Overall, 163 managers from 82 districts enrolled in the trial (figure 1) and contributed data for the primary analysis, representing 61% of Uganda’s 135 districts (appendix p 10). All managers were Ugandan, and 149 (91%) were male. Characteristics of study districts are summarised in table 1.

Intervention delivery began on Dec 6, 2017 (southwest), and March 28, 2018 (east and east-central), and continued until Feb 2, 2022. Average attendance at biannual in-person meetings was 99% in the southwest region and 83% in the east and east-central regions (appendix p 11). Due to COVID-19 restrictions, the meetings 2·5 years post-randomisation were delayed by 4 months and one meeting was held virtually (the 2-year east and east-central meeting). Staff provided data dashboards to all intervention groups at each meeting. Although staff provided training and access to the SMS platform at baseline, only 23 (53%) of 43 districts used the platform; average duration of use was 7·8 months (range 2–26).

**Figure 2:**
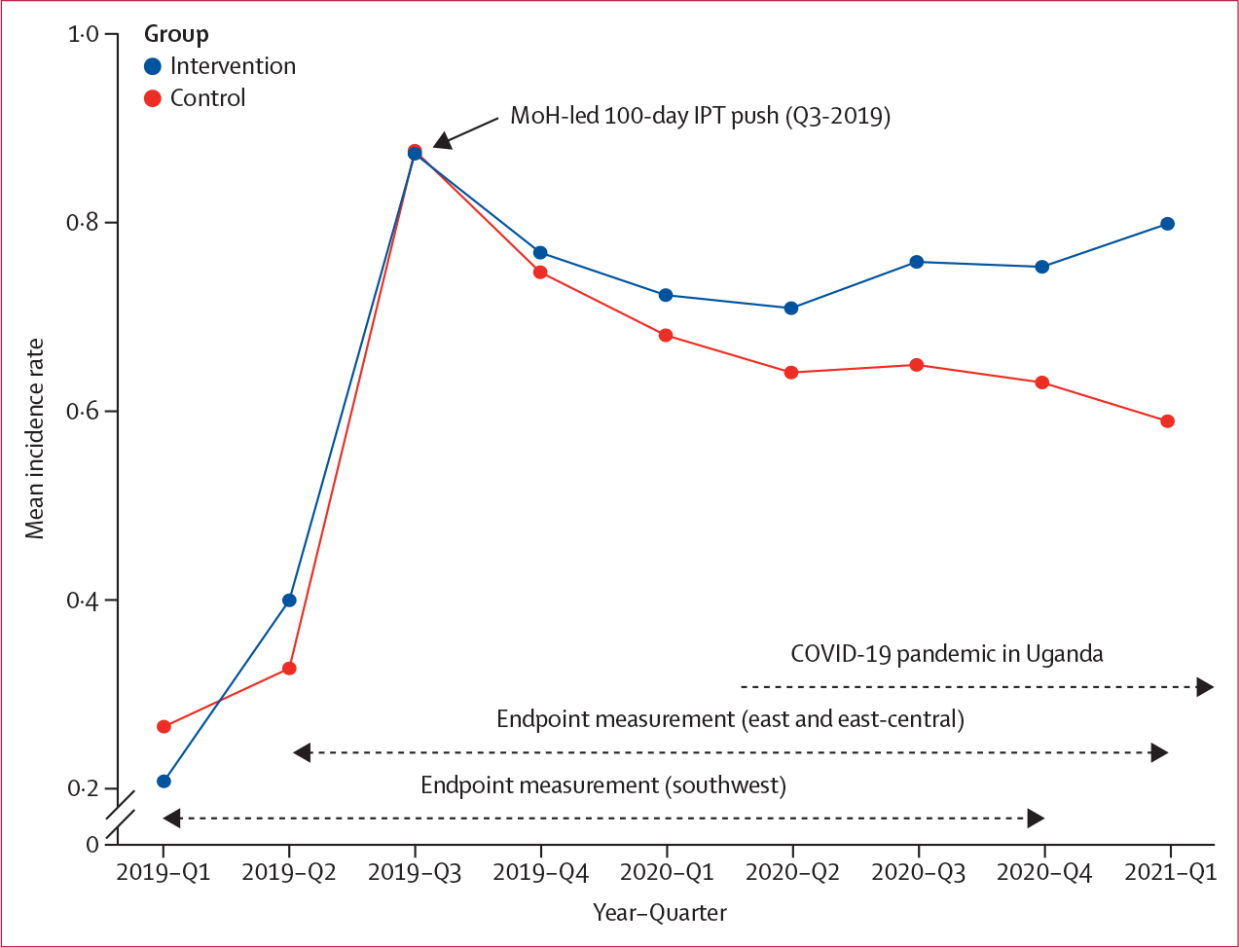
IPT initiation incidence rates over time in intervention versus control groups IPT=isoniazid preventive therapy. MoH=Ministry of Health.

Over the 2-year measurement period, the incidence of IPT initiation among adults with HIV was 0·74 starts per person-year (95% CI 0·59–0·88) in the intervention group and 0·65 starts per person-year (0·55–0·75) in the control group (incidence rate ratio [IRR] 1·14, 95% CI 0·88–1·46; p=0·16; table 2). In prespecified analyses that excluded the 100-day IPT push (Q3–2019), the incidence of IPT initiation among adults with HIV was significantly higher in intervention than control districts: 0·32 (95% CI 0·26–0·38) versus 0·25 (0·21–0·29) starts per person-year, respectively (IRR 1·27, 95% CI 1·00–1·61; p=0·026; figure 2). The incidence of IPT initiation stratified by sex was higher in the intervention group than the control group when excluding Q3–2019 among men (IRR 1·27, 95% CI 1·03–1·56; p=0·012) and women (1·21, 0·94–1·55; p=0·068). Similar trends were observed in all regions (table 2).

Over 2 years, the mean cumulative incidence of IPT initiation among adults in HIV care was 68% (95% CI 58–77) in the intervention group and 65% (59–71) in the control group (adjusted risk ratio [aRR] 1·04, 95% CI 0·88–1·22; p=0·33). In analyses excluding Q3–2019, cumulative incidence of IPT initiation was significantly higher in the intervention group (40%, 95% CI 35–45) than the control group (34%, 30–38); aRR 1·17 (95% CI 1·0–1·4; p=0·038).

Among 801 adults selected for chart review from eight intervention and eight control clinics (50–51 charts per clinic) in the southwest region, 715 (89%) completed IPT within 9 months of isoniazid start. There was no significant difference in IPT completion by trial group: 366 (91·3%) of 401 participants in the intervention group and 349 (87·3%) of 400 in the control group (aRR 1·03, 95% CI 0·98–1·08; p=0·11, adjusting for sex, age, and timing of IPT initiation). Among adults who initiated IPT before Q3–2019, a significantly greater proportion completed IPT in the intervention group (92·8%, 95% CI 89·0–96·5) versus the control group (84·9%, 80·0–89·9); aRR 1·09 (95% CI 1·02–1·17), p=0·0074. Among adults who initiated IPT during the 100-day IPT push, there was no significant difference in completion by group: 89·9% intervention versus 92·0% control (aRR 0·98, 95% CI 0·92–1·04; p=0·23).

The incidence of tuberculosis disease among people with HIV at the district level was 1·61 cases per 100 person-years in the intervention group versus 1·57 cases per 100 person-years in the control group (IRR 1·02, 95% CI 0·86–1·23; p=0·39). There was no difference in tuberculosis incidence when Q3–2019 was excluded.

At baseline, 52 managers in intervention and 51 in control districts responded to the survey regarding IPT and tuberculosis prevention. At 1-year follow-up, 72 managers in the intervention group and 77 in the control group responded, and at 2 years, 54 and 52 responded, respectively. From baseline to year 1, there were significantly greater average increases in familiarity with IPT and in knowledge of IPT efficacy among intervention versus control districts (table 3).

In FGDs, intervention-group managers reported improved communication among stakeholders, which helped them identify where training or mentorship of junior colleagues was needed. Improved communication and a sense of empowerment motivated managers to identify root problems in the supply chain and push for better logistics management. In some districts, this meant moving medication from areas with surplus stocks to places with greater need. Intervention-group managers reported that mini-collaboratives helped break down district silos, creating a collegial environment and promoting greater teamwork and regional cooperation. They reported that the dashboards provided motivation to begin tracking and reviewing data consistently. They reported that leadership and management training promoted use of local data to create clear IPT targets, with explicit timelines, and helped them organise and focus their efforts and feel empowered to use data to make decisions. Lastly, they reported that the intervention improved other health activities, such as greater regional cooperation in contact tracing and treatment for active tuberculosis.

In contrast, control-group managers reported challenges managing personnel, including lack of teamwork and morale among frontline providers. They also reported an interest in receiving regular feedback on progress with tuberculosis prevention and learning from well performing districts, and noted challenges with regular data review and communication within their districts and regionally, particularly during the COVID-19 pandemic (appendix p 12).

Intervention start-up cost US$27 122 over 6 months post-randomisation in the three regions. This amount included $12 183 in stakeholder engagement, protocol development, and training of study staff; $11 334 to convene the first mini-collaborative meeting; and $3605 to develop, install, and conduct training for the SMS platform. Estimated total cost of the intervention was $83 508 during year 1 and $62 384 in each subsequent year; of this total, holding biannual mini-collaborative meetings in year 1 with international business consultants cost $33 045 per year. Estimated cost of holding mini-collaborative meetings in subsequent years with local business consultants would have cost $11 638 per year. Intervention cost per district was $3716 in year 1, and $1433 in each subsequent year. Intervention cost per additional person who initiated IPT was $70·45 overall and $23·21 after excluding Q3–2019.

## Discussion

In this cluster randomised trial that enrolled mid-level managers from over half of Uganda’s districts, an intervention that provided structured leadership and management training and facilitated subregional collaboration and routine data feedback resulted in increased IPT knowledge, and improved within-district communication and inter-district collaboration. Although overall IPT initiation rates were not significantly higher with the mid-level manager intervention, rates were significantly higher compared with control after excluding the massive MoH-led 100-day IPT push in both trial groups. The higher rates were sustained during the COVID-19 pandemic, suggesting benefits of leadership and management training and collaboration for mid-level health managers who operate at the nexus of guidelines and implementation.

Experimental evidence for interventions to increase IPT implementation in low-income countries has been limited to date. Despite strong evidence supporting IPT,^1,2^ low IPT use among people with HIV has been a persistent challenge globally for over a decade.^3^ Although increasing IPT initiation rates have been described in some settings in sub-Saharan Africa over the past few years,^3^ uptake has been variable, with a wide range of strategies applied and largely assessed via observational studies. Strategies have included operational guidance, provider training and mentorship, isoniazid stock support, changes to how isoniazid is delivered, and HIV–tuberculosis service integration.^26,27^

In our trial, we sought to determine if an intervention centered on district-level health managers could increase IPT initiation among people with HIV compared with standard practice, starting at a time (2017–18) in which less than 2% of people with HIV in Uganda had received IPT.^10^ However, our outcome measurement period coincided with the massive MoH-led 100-day IPT push and the COVID-19 pandemic. The Q3–2019 push, with improved isoniazid supply and the US Government implementing partner investment in collection and transmission of weekly data from push sites, achieved impressive increases in IPT use in both trial groups, showing what can be done with concentrated efforts: of over 500 000 people with HIV initiated on IPT from October, 2018, to October, 2019, in Uganda, more than 65% (343 674) of initiations occurred during this push.^10^ In contrast, our intervention focused on long-term capacity and skills building among health managers. The skills building of managers in the intervention group led to sustained increases in IPT initiation rates in contrast to control after the 100-day push. The intervention also led to higher rates of IPT use during the disruptive impact of COVID-19 from 2020 onward. The COVID-19 pandemic led to a decline in people with HIV initiated on tuberculosis preventive therapy globally in 2020 compared with 2019.^28^ Our findings suggest that the intervention might have helped managers leverage the intensive 100-day push and cope with the COVID-19 pandemic, to sustain high rates of IPT. Ultimately, the 6 percentage point higher average cumulative incidence of IPT initiation in intervention versus control after excluding the 100-day push translates to approximately 29 000 more people with HIV starting IPT in the study districts when extrapolated to the population level. This absolute increase in IPT initiation is likely to translate into fewer tuberculosis cases, and ultimately mortality reduction, for people with HIV in the trial regions.

Our intervention combined several components designed to improve IPT initiation. Health system supervisors are often clinicians with little or no formal leadership or management training.^29^ Several studies have found associations between leadership and management skills of health system supervisors and health outcomes, including childhood vaccination and primary care delivery.^30,31^ The leadership and management training we provided might have contributed to intervention effectiveness by improving competence in these areas and offering concrete tools to apply these skills. Facilitation of collaboratives by Ugandan expert coaches probably contributed to improved understanding of IPT effectiveness and provided reinforcement of guidelines from trusted sources. Biannual meetings provided a venue for managers to compare progress with peers, reinforce a shared objective, and discuss best practices. Routine data review through “audit and feedback” has been shown to improve health-care outcomes.^32^ In a systematic review of audit and feedback intervention trials,^32^ factors associated with more effective interventions included provision of feedback more than once, presentation via both verbal and written formats, receiving data from colleagues or supervisors, and explicit action plans, all of which were part of our intervention.

The focus on leadership and management capacity building among district-level managers and collaboration between peer managers might have created an environment to speed dissemination of innovation and leverage social comparisons. Indeed, intervention-group managers reported greater motivation, collaboration, empowerment, and familiarity with, and knowledge of, IPT than the control group. Interestingly, one recent trial that randomised nurse supervisors overseeing primary care clinics in South Africa also used an intervention that relied on biannual collaboratives and review of routinely collected clinical data to achieve significant increases in IPT initiation.^33^ In this South African trial, collaboratives were formed between nursing supervisors and healthcare staff at the clinics they oversaw, and capacity building focused on quality improvement. In contrast, our intervention focused on mid-level managers, who operate upstream of multiple clinics and frontline providers, suggesting that intervening among managers offers one way of influencing provider behaviour. Whether this training had impacts on other health domains beyond IPT remains an important question moving forward.

This trial’s intervention resulted in significant increases in IPT completion before Q3–2019 without significant differences in HIV-associated tuberculosis incidence between the intervention group and the control group. Although seemingly counterintuitive given the efficacy of IPT in reducing tuberculosis disease, the trial might have been underpowered to detect differences in HIV-associated tuberculosis incidence. Furthermore, improved tuberculosis case-finding, including screening before IPT, might have led to increased case detection in intervention districts in the context of higher IPT initiation rates. Increased completion of IPT in intervention districts among people with HIV who initiated isoniazid before Q3–2019 might have been a result of expert coaching on the importance of isoniazid adherence during mini-collaborative meetings or a secondary effect of leadership and management training.

This study has limitations. First, multiple nationwide secular events occurred during the trial and impacted study outcomes. To account for these events, which affected both intervention and control groups, we conducted prespecified sensitivity analyses in accordance with CONSERVE 2021 recommendations.^23^ These analyses demonstrated intervention effectiveness after accounting for or despite these events. Second, given the large scale of the trial, we cannot exclude potential contamination of intervention effects due to contact between intervention and control group managers. However, this contamination, if it occurred, would bias trial results towards a null effect, suggesting that the intervention effects observed provide a conservative estimate of effectiveness. Finally, health outcomes relied on data from the two largest clinics in each district; whether the intervention affected outcomes at smaller clinics is unknown. Despite these limitations, this study adds important findings to the literature on effective interventions to improve IPT implementation and shows that engaging mid-level health supervisors can improve health outcomes on a large scale.

In conclusion, an intervention among mid-level health managers in Uganda that provided structured leadership and management training and facilitated subregional collaboration and routine data feedback resulted in significant increases in IPT initiation and completion, after accounting for several nationwide, secular events, compared with standard practice.

## Supplementary Material

1

## Figures and Tables

**Table 1:** Characteristics of districts participating in intervention and control groups

	Intervention	Control

Number of clusters	7	7
Number of districts	43	39
Number of managers	86	77
District health officers	43	38
District tuberculosis supervisors	43	39
Sex of managers		
Male	78(91%)	71 (92%)
Female	8 (9%)	6 (8%)
Regions		
Southwest	13	12
East	12	11
East-central	18	16
Number of districts per randomisation cluster	5(5–6)	5(5–6)
Number of adults in active HIV care at the district level	5182 (2340–8346)	3456 (1949–8260)
Number of adults in active HIV care at the two largest clinics in each district	2099 (1270–3304)	1897 (1181–3378)
HIV prevalence: proportion of adults in HIV care among the total adult population in each district	4·7% (1·8–6·2)	2·3% (1·7–5·0)
IPT uptake*: proportion of adults in HIV care at the two largest clinics who had received IPT in the quarter immediately preceding the measurement period	1·8% (0·4–5·2)	2·2% (0·6–5·3)
Active tuberculosis prevalence†: proportion of adults with tuberculosis disease diagnosis among adults in active HIV care at the district level in the quarter immediately preceding the measurement period	0·4% (0·3–0·6)	0·3% (0·2–0·4)

Data are n, n (%), or median (IQR). Randomisation was conducted within pairs of clusters matched on region, number of adults in HIV care, presence of large urban centres, and presence of a community that had participated from 2013 to 2017 in the SEARCH universal HIV test-and-treat trial. IPT=isoniazid preventive therapy.

* Missing data on four clinics.

† Missing data on two districts.

**Table 2:** IPT initiation rate by trial group, overall and after excluding the 100-day IPT push occurring in Q3–2019, with subanalyses by sex and region

	IPT initiation per person-year (95% CI)		Incidence rate ratio (95% CI)	p value*
	Intervention	Control		

**Whole 2-year period**				
Overall	0·74 (0·59–0·88)	0·65 (0·55–0·75)	1·14 (0·88–1·46)	0·16
By sex				
Men	0·78 (0·64–0·92)	0.69 (0·58–0·79)	1·13 (0·89–1·44)	0·15
Women	0·68 (0·54–0·80)	0·63 (0·52–0·73)	1·08 (0·83–1·41)	0·23
By region				
Southwest	0·65 (0·41–0·90)	0·71 (0·50–0·92)	0·92 (0·57–1·48)	0·35
East-central	0·79 (0·62–0·95)	0·72 (0·58–0·86)	1·09 (0·81–1·46)	0·27
East	0·78 (0·42–1·15)	0·45 (0·23–0·67)	1·75 (0·89–3·44)	0·048
**Excluding Q3-2019 (100-day IPT push)**				
Overall	0·32 (0·26–0·38)	0·25 (0·21–0·29)	1·27 (1·00–1·61)	0·026
By sex				
Men	0·33 (0·28–0·38)	0·26 (0·22–0·30)	1·27 (1·03–1·56)	0·012
Women	0·30 (0·24–0·35)	0·25 (0·21–0·29)	1·21 (0·94–1·55)	0·068
By region				
Southwest	0·31 (0·20–0·42)	0·29 (0·19–0·39)	1·07 (0·66–1·75)	0·38
East-central	0·32 (0·23–0·40)	0·25 (0·21–0·29)	1·25 (0·92–1·72)	0·073
East	0·34 (0·27–0·40)	0·20 (0·10–0·29)	1·71 (1·00–2·90)	0·024

IPT=isoniazid preventive therapy. Q3-2019=third quarter of 2019.

* One-sided test of the null hypothesis that the trial intervention did not improve IPT initiation among adults in HIV care.

**Table 3:** Comparison of quantitative survey responses in intervention versus control groups

	Intervention: mean scores			Control: mean scores			Difference in score changes: intervention *vs* control (95% CI)	p value*
	Baseline	Year 1	Change (95% CI)	Baseline	Year 1	Change (95% CI)		

How familiar are you with IPT?†	3·72	4·73	+0·52 (0·03 to 1·0)	4·05	4·09	+0·05 (−0·46 to 0·55)	+0·47 (0·44 to 0·80)	0·0034
How strong is the evidence that isoniazid prevents active tuberculosis in HIV-infected patients?‡	3·75	4·38	+0·63 (−0·1 to 1·36)	4·14	4·18	+0·05 (−0·63 to 0·72)	+0·59 (0·06 to 1·12)	0.015
How difficult is it for providers in this district to add isoniazid to standard care for HIV-positive people in order to prevent tuberculosis?§	2·38	2·23	−0·15 (−0·99 to 0·69)	2·52	2·15	−0·36 (−1·31 to 0·58)	+0·21 (−0·26 to 0·69)	0·183
How hard is it to influence changes in practice among frontline providers around tuberculosis management?§	2·78	2·42	−0·37 (−1·0 to 0·27)	2·68	2·45	−0·23 (−0·86 to 0·41)	−0·14 (−0·62 to 0·35)	0·282

The left column shows the survey questions, which were scored on a Likert scale with a range of 1–5. Responses to 1 and 5 scores are listed in the footnotes. IPT=isoniazid preventive therapy.

* One-sided p value.

† 1=no knowledge of IPT, 5=high knowledge of IPT.

‡ 1=very weak, 5=very strong.

§ 1=very easy, 5=very difficult; declining score (negative change) indicates decreasing difficulty (ie, increasing ease) for these questions.

## Data Availability

A complete de-identified dataset sufficient to reproduce the primary study findings will be made available upon request to the corresponding author, following approval of a concept sheet summarising the analyses to be done.

## Research in context

### Evidence before this study

Tuberculosis is the leading cause of death for people living with HIV worldwide. Multiple randomised trials have demonstrated that isoniazid preventive therapy (IPT) reduces the risk of active tuberculosis by approximately 40–60% for people with HIV, in addition to tuberculosis risk reduction from antiretroviral therapy. For over a decade, international guidelines have recommended IPT for people with HIV, yet global use of tuberculosis preventive therapy has remained low, particularly in sub-Saharan Africa. We searched PubMed on Nov 1, 2021, for literature on interventions to increase IPT implementation among people with HIV in sub-Saharan Africa, using the search terms (“isoniazid preventive therapy,” OR “TB preventive therapy,”) AND “HIV” AND “implementation” AND “Africa,” without language or date restrictions. Although several studies described increasing IPT initiation rates in some settings in sub-Saharan Africa over the past few years, reported uptake has been variable, with a wide range of strategies applied and largely assessed via observational studies. In published observational studies, strategies to increase IPT implementation have included operational guidance, provider training and mentorship, isoniazid stock support, changes to how isoniazid is delivered, and HIV–tuberculosis service integration. One recent cluster randomised trial in South Africa tested an intervention among nurse supervisors overseeing primary care clinics that relied on biannual collaboratives and review of routinely collected clinical data to achieve substantial increases in IPT initiation. In the trial, collaboratives were formed between nursing supervisors and health-care staff at the clinics they oversaw, and capacity building focused on quality improvement. Several ongoing trials, with methods, but not yet results, available in the literature are also examining interventions to increase IPT use with a focus on frontline health-care workers. However, to our knowledge, no experimental studies have evaluated interventions among health system middle managers to increase IPT implementation in sub-Saharan Africa.

### Added value of this study

In this cluster randomised trial that enrolled mid-level health system managers from 61% of Uganda’s districts, an intervention that provided structured leadership and management training and facilitated subregional collaboration and routine data feedback resulted in increased IPT knowledge and improved within-district communication and inter-district collaboration. The trial launched at a time (2017–18) when less than 2% of people with HIV had received tuberculosis preventive therapy in Uganda. Although overall IPT initiation rates were not significantly higher with the mid-level manager intervention, rates were significantly higher compared with control after excluding a massive Ministry of Health-led 100-day IPT push tied to a financial contingency, in both groups of the trial. The higher rates were sustained during the COVID-19 pandemic, suggesting benefits of targeted leadership and management training and collaboration for mid-level health managers who operate at the nexus of guidelines and implementation.

### Implications of all the available evidence

To date, published evidence for interventions to improve implementation of tuberculosis preventive therapy among people with HIV has largely focused on intervening among frontline health-care workers and at the clinic–patient interface. However, mid-level health managers oversee implementation of guidelines at the subnational (eg, district or province) level in many sub-Saharan African countries. Our intervention is novel in that it engaged mid-level managers to promote evidence-based health recommendations. Our findings suggest that interventions centred on mid-level health managers can improve IPT implementation on a large, subnational scale, and merit further exploration to address key public health challenges for which strong evidence exists but implementation remains suboptimal.
