# Supplementary material for: A mid-level health manager intervention to promote uptake of isoniazid preventive therapy among people with HIV in Uganda: a cluster randomised trial
Source: Lancet HIV. Author manuscript; Available in PMC 2022 Oct 6. (PMC9536151; doi:10.1016/S2352-3018(22)00166-7)
Supplement: 1 [file NIHMS1833570-supplement-1.pdf]

THE LANCET
HIV

## Supplementary appendix

This appendix formed part of the original submission and has been peer reviewed. We post it as supplied by the authors.

Supplement to: Kakande E, Christian C, Balzer LB, et al. A mid-level health manager intervention to promote uptake of isoniazid preventive therapy among people with HIV in Uganda: a cluster randomised trial. *Lancet HIV* 2022; published online July 28.
https://doi.org/10.1016/S2352-3018(22)00166-7.

**Appendix**

**Title**: A mid-level health manager intervention to promote uptake of isoniazid preventive therapy in Uganda: a cluster randomized trial

Table of Contents

1) **Business Leadership and Management Training Summary**: Overview of annual business leadership and management training content in the intervention districts.

2) **Modification Timeline**: Secular Events during the trial with resulting modifications to measures and analyses prior to unblinding (in accordance with CONSERVE 2021 recommendations)

3) **Statistical Analysis Plan**

4) **Supplementary Table 1**: Summary of district data and participant subgroups included in the primary and secondary analyses, by trial arm.

5) **Supplementary Figure**: Map of intervention and control districts in Uganda.

6) **Supplementary Table 2**: District manager attendance at biannual meetings.

7) **Supplementary Table 3**: Qualitative interview quotes.

**Business Leadership and Management Training Summary.** Overview of annual business leadership and management training content in the intervention districts.

# 'One Day MBA' Curriculum

---

## Part 1: Kotter's 8 Step Model

**What is Kotter's 8 Step Model for Change:** This model was developed by a Harvard Business Professor, John Kotter, on the basis of research of 100 organizations. This model is taught by most major international business schools and many global corporations including Ericsson, Pepsi and Coca-Cola have successfully used it to create change.

**Key to Kotter's Model:** Change requires leadership *and* management

- **Management**: Planning and budgeting, organization and staffing, controlling and problem solving
- **Leadership:** establishing direction, aligning people, be motivating and inspiring

### 8 Steps of Kotters Method:

1. **Establish a sense of urgency**
2. **Create our guiding coalition with**
    a. A shared objective
    b. Trust
    c. The right people – power, expertise, credibility
3. **Develop our Change Vision**
    a. Bold but achievable
    b. Paints a vivid picture of the future
    c. Appeals to health workers' hearts (and minds)
    d. Is specific enough to help individuals make decisions independently and weigh trade-offs
    e. Is flexible enough to adapt to changing conditions
    f. Is easy to communicate quickly – in 60 seconds
4. **Communicate our vision for buy-in**
    a. Put our vision everywhere
5. **Empower Broad-based Action**
6. **Generate Short-term wins**
    a. What can you do in 10 minutes, 10 days, 10 weeks?
7. **Never Let Up**
8. **Incorporate change into the culture**

**Incorporating these steps into your work as DHOs and DTLSs in improving INH uptake by eligible people living in your district:** What can you do in the next 10 minutes, or 10 days, or 10 weeks to make changes in the prescribing of INH in your district?

# <u>Part 2</u>: Objective Key Results (OKRs)

**What are Objective Key Results (OKRs)?**

- Frame work for defining objectives and tracking their outcomes
- Established management technique for recurring planning and progress reporting
- It's way to run your life, team or organization
- OKRs are ***not*** initiatives – Initiatives are what we are going to do to reach our goal (Tasks, activities, projects) while objectives outline what we want to achieve and key results are how we measure progress.

**An Objective** is a description of a goal to be achieved in the future. An Objective sets a clear direction and provides motivation. What do we want to achieve?

An ***Objective*** should be:

- **Directional**
- **Ambitious**
- **Aligned**
- **Time-bound**

**Key Results –** are the "how" - A Key Result shows you how you're progressing towards your Objective. How are we going to measure our progress?

A ***Key Result*** should:

- **Make the Objective achievable**
- **Be measurable**
- **Not a task/to do**

<u>Successful OKRs are:</u>

- Quantitatively trackable through measurable key results
- Looked at frequently (daily, weekly, monthly)
- Stretch goals that are a challenge to achieve (50%-70% chance of achievement)

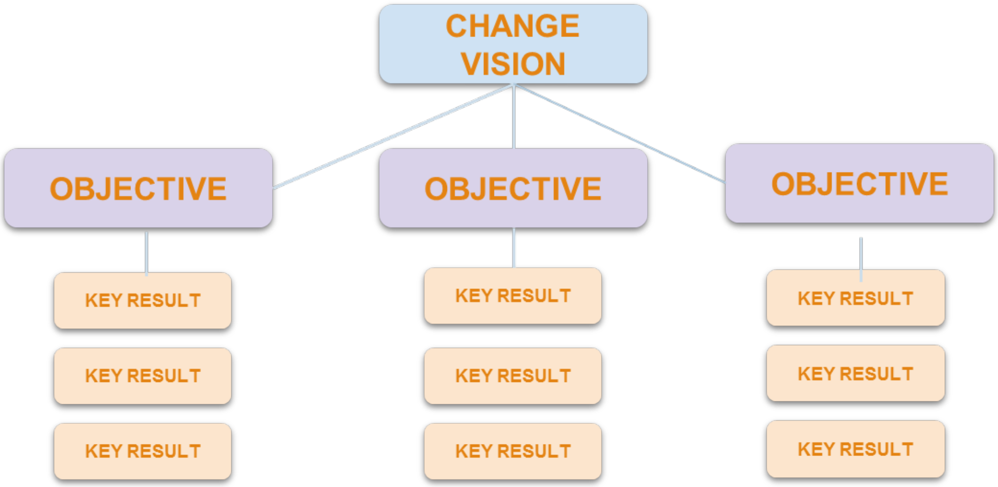


**How to use ORKs in your work:** List three main objectives for your change vision and three Key Results (measurable outcomes) for each of these objectives. Track these key results as progress toward your objectives and overall change vision.

SEARCH
SUSTAINABLE EAST AFRICA RESEARCH IN COMMUNITY HEALTH

# <u>Part 3</u>: Stop. Start. Continue.

**What is Stop. Start. Continue?**

- A simple and effective way for teams to **reflect** and **decide** on **what actions should change** as they move forward
- Borrowed from the "**Agile**" project management method widely adopted by most modern software companies

**Why is it important to Reflect?**

- Gives teams an **opportunity to review** how they are doing and **identify improvements** they can implement in the future
- Makes it easier for teams to **clarify issues**, **weight the impact** of ideas, and reach a **consensus based on shared priorities**
- Is very **action orientated** and provides **momentum** and **energy** for the team. Each item on the list results in behavioral change.
- Empowers teams to **continuously improve** the way they work

1. **Start** – activities are those things the team will begin doing in the next cycle.
   **These are activities that may:**
   - improve processes
   - reduce waste (time)
   - have a positive impact on the way the team operates

2. **Stop** – looks back at the previous cycle of the project to identify which things didn't work and should cease
   **Consider activities that:**
   - are inefficient
   - waste time and/or resources
   - have a negative impact on the way people feel or the way things work

3. **Continue** – identifies things that worked in the previous cycle and need to continue to be part of the team's core activities

**Think about activities that:**

- the team has tried and were successful but are not yet part of common practice. Once the activities are part of the way things are done, add them to procedure manuals and checklists and remove from this list.

**How to use the Start Stop Continue toolkit to evaluate your district's performance**: Looking back at your objectives you set last quarter and the plans you put into action to achieve them, what are some activities that you want to Start, Stop or Continue to improve your performance?

SEARCH
SUSTAINABLE EAST AFRICA RESEARCH IN COMMUNITY HEALTH

**Modification Timeline due to Extenuating Circumstances (i.e., secular events)**

| Extenuating circumstance in Uganda | Date identified | Trial Modification | Dates when circumstances took effect on trial | Number of clusters enrolled before extenuating circumstance | Number of clusters enrolled after extenuating circumstance | Number of clusters that completed the trial before the extenuating circumstance |
|---|---|---|---|---|---|---|
| Nationwide isoniazid (INH) Stock Outs | December 2017 | To test our intervention in a context where INH supply was not the limiting factor, we defined our endpoint measurement period over two years starting in 2019 (Q1-2019: Southwest and Q2-2019: East/East-Central): modified protocol, version April 1, 2021 prior to unblinding | December 2017 – Q4-2018 (Southwest) and Q1-2019 (East/East Central) | 79 | 4 | 0 |
| Quarter 3-2019 “100-Day IPT Push” | July 2019 | To better understand the trial intervention’s impact on IPT initiation independent of the “100-day IPT push”, we conducted pre-specified secondary analyses that excluded Q3-2019: modified protocol, version April 1, 2021 prior to unblinding | July – September, 2019 | 83 | 0 | 0 |
| Nationwide COVID-19 pandemic lockdown | April 2020 | In Q2-2020, the Ugandan President ordered a nationwide COVID-19 lockdown. We conducted pre-specified sensitivity | March 2020 – end of trial | 83 | 0 | 0 |

| | | | | | | |
|---|---|---|---|---|---|---|
| | | analyses that evaluated outcomes pre/post-lockdown. Statistical Analysis Plan (SAP) Version 1.0 was locked on July 18, 2021 prior to unblinding and effect estimation. | | | | |

**Statistical Analysis Plan**.
Accessible at the following url: https://arxiv.org/abs/2111.10467

**Supplementary Table 1.** Summary of district data and participant subgroups included in the primary and secondary analyses by trial arm.

| Endpoint | Intervention (43 Districts) | Control (39 Districts) |
|---|---|---|
| **IPT initiation incidence rate (primary)** | All Districts: 127,021[a] adults in active care in the two largest clinics/district | All Districts: 122,784[a] adults in active care in the two largest clinics/district |
| **HIV-associated TB incidence** | All Districts: 247,284[b] adults in active HIV care (all clinics/district) | All Districts: 234,700[b] adults in active HIV care (all clinics/district) |
| **IPT Completion** | *Southwest Region only*: Chart Review of randomly-selected patients (PWH, ≥15, who initiated IPT) at 8 facilities (N=401) | *Southwest Region only*: Chart Review of randomly-selected patients (PWH, ≥15, who initiated IPT) at 8 facilities (N=400) |
| **Quantitative Survey** | All Districts<br>Number of managers completing survey: Baseline=52; Year 1[c]=72; Year 2[c]=54 | All Districts<br>Number of managers completing survey: Baseline=51; Year 1[c]=77; Year 2[c]=52 |
| **Qualitative Interviews** | All Districts<br>Focus Group Discussions (FGDs): N=4 FGDs<br>Southwest: Year 1 (7 managers); Year 2 (11 managers)<br>East/East-Central: 2 FGDs in Year 1 (9 managers each) | All Districts<br>Key Informant Interviews (KIIs): N=23 KIIs<br>Southwest: Year 1 (4 managers); Year 2 (5 managers)<br>East/East-Central: Year 1 (6 managers); Year 2 (8 managers) |
| **Costing** | All Districts<br>Interviews with study staff and Manager costing surveys (18 managers) | Not applicable |

[a]Average active HIV care size in two largest clinics/district over follow-up
[b]Total active HIV care size (all clinics/district) at baseline
[c]Years post-randomization

**Supplementary Figure**. Map of intervention and control districts in Uganda.

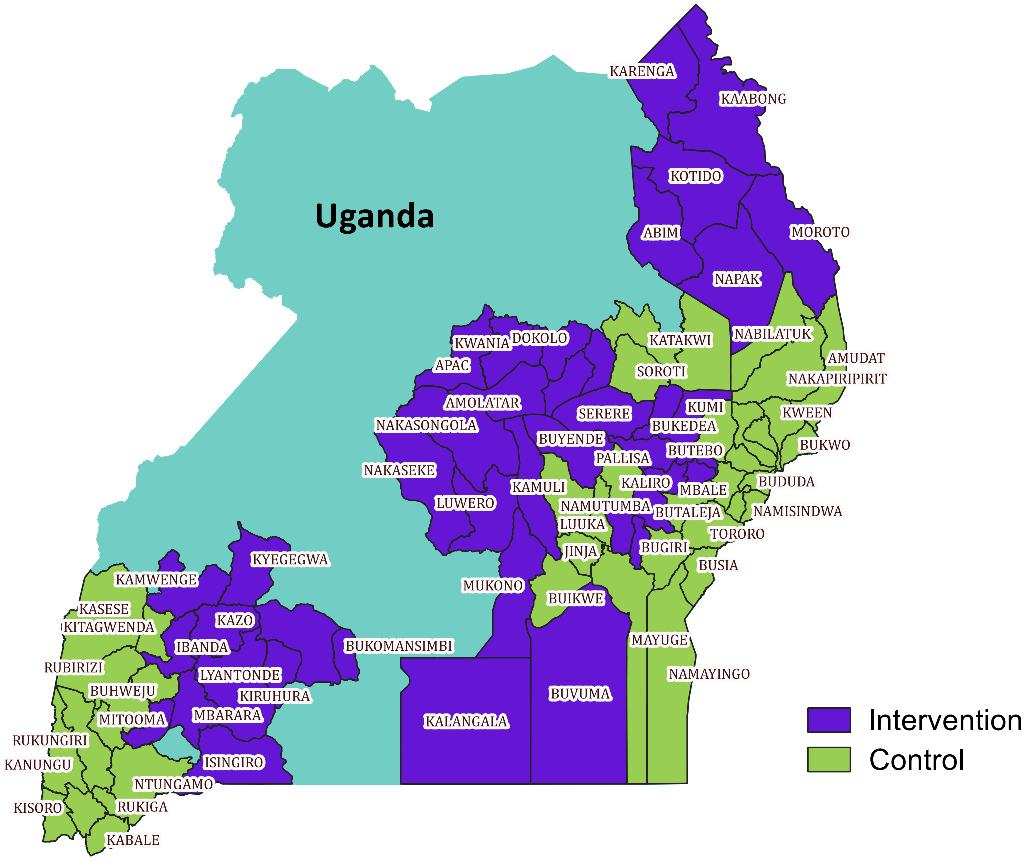

**Supplementary Table 2.** Intervention-district mini-collaborative meeting participation, in which at least one manager representing a district attended.

| **Region** | **Intervention Mini-collaborative Meeting Timepoint** | | | | | | | |
|---|---|---|---|---|---|---|---|---|
| | **Initial Meeting** | **2-3 months** | **6 months** | **12 months** | **18 months** | **24 months** | **30 months** | **36 months** |
| **Southwest** (N=13 districts) | 13/13 *(100%)* | 13/13 *(100%)* | 12/13 *(92%)* | 13/13 *(100%)* | 13/13 *(100%)* | 13/13 *(100%)* | 13/13 *(100%)* | 13/13 *(100%)* |
| **East & East-Central** (N=30 districts) | 25/30 *(83%)* | 27/30 *(90%)* | 21/30 *(70%)* | 22/30 *(73%)* | 27/30 *(90%)* | 7/30* *(23%)* | 23/30 *(77%)* | N/A** |

*Virtual mini-collaborative meeting due to COVID-19 lockdown in Uganda
**Postponed due to COVID-19

**Supplementary Table 3.** Sample quotations from Focus Group Discussions and Key Informant Interviews.

| Domain | Focus Group Discussions (Intervention Group Managers) | Key Informant Interviews (Control Group Managers): Comparator quotes related to Domain |
|---|---|---|
| Impact of Study | • *"These meetings have broken the barriers; they have broken through those walls. ... we realize that these diseases do not know borders, they don't know that district 1 starts and ends here, district 7 ends here while district 5 ends here but rather, this is a more regionalized group and if we are going to break through, we need to work together."*<br><br>• *"For those of us who were in the study and what we are now, at least there is a very big difference that is: the accessibility of our clients has improved, our capacity to use our own data improves every other time we meet for the collaborative meetings because we have the trigger."*<br><br>• *"We start to ask ourselves questions like; how can we work with Ips [Implementing Partners]? How can we work to ensure that the stocks of INH are there to serve the people, how can we make our health facilities qualified to have the stocks that INH has? So, you find that a person uses their own resources, the knowledge in terms of the gap and you find that you can add a step and do better."* | • *"The main challenge is that we have not built enough capacity, there's need for capacity building for our staff and all those involved to appreciate IPT."*<br><br>• *"Another way the study can get to inform us is to tell us how other districts are performing so that we can have study tours in the best performing districts and actually learn from them."* |
| Impact of Data Dashboards | • *"There is no shaming or naming that is equivalent to the evidence of data. You just flash it there and you see that [your district] is the first from the bottom and you say eeh…"*<br><br>• *"For me it was a wakeup call because when we look at our initiation of clients to IPT, we were doing poorly so when I was in that meeting I was majorly challenged … I think over time we are moving in the positive direction."*<br><br>• *"The aspect of data and the progressive report, for me as a manager,... I found it to be a handy* | • *"I have not done a study to see how many patients go without INH, but I can get that."* |

| | | |
|---|---|---|
| | *tool, to realign my workforce and to keep telling them that this is the target, and that we are way below, or I give them a tap on the shoulder that "you see, we are now at 95%, keep it there because that is what we want to be." You see having data is very important and when you use it, you become a very objective manager. You cease leading impulsively or from as sentimental approach, you are more objective."* | |
| Impact of leadership and management courses | • *"If we do not put it in terms of business, we shall not know what we expect. If you are in the ART clinic, you must know that this day, you should expect 30 people, do you have their drugs available with you, what have you done? So, you have to go around and look for the drug as you prepare for the next day."*<br><br>• *"What I have learnt through these collaborative meetings, what I have learnt specifically is to draw a work plan on how to implement IPT whereby we look at what you can do in a specific duration of time."*<br><br>• *"We are talking about the activities that we set out to do, we had a work plan and there were targets. Given the targets, how much were we able to achieve and everybody presents. It is the data now to judge and I find that very informative."* | • *"The support we need really are logistics. We have the human resources to do the work and the human resource has the knowledge to do the work but the logistics side is the problem."*<br><br>• *"Currently it is more of sacrifice, there's no benefit a [manager] gets from implementing TB prevention […] I don't feel motivated."* |
| Impact on non-IPT-related health activities | • *"Now what has happened is that we have twinned the two, the one from the IP (Implementing Partner) and the one from the government (the DTLS [District TB Supervisor]). So, they will be working as teams: ours of course has the mandate of the district but the other one has more resources. So, we see more resources from the IP towards this prevention aspect that is why we're even scaling up in other health facilities beyond the ones that we have in this study."* | • *"It is only COVID-19 that has come up with online and virtual modes of communication, which we have not yet been embraced, […] we still use our usual modes of communication."* |
